# Supplementary material for: Impact of Irradiation on the Adhesive Performance of Resin-Based Dental Biomaterials: A Systematic Review of Laboratory Studies
Source: Materials (Basel). 2023 Mar 24;16(7):2580. doi: 10.3390/ma16072580 (PMC10095157; doi:10.3390/ma16072580)
Supplement: Supplementary file 1 [file materials-16-02580-s001.zip › materials-2298574-supplementary.pdf]

Table S1: Search strings used for the searches in five electronic databases

Electronic database	Search string	Number of records identified
Cochrane Library	(Tooth OR teeth OR enamel OR dentin) AND (Radiotherapy OR irradiation OR radiation therapy OR radiation treatment OR oral cancer treatment OR ionizing radiation) AND (Dental Bonding OR bond strength OR marginal discoloration OR microleakage OR debonding OR interfacial fracture toughness OR marginal adaptation) AND (In Vitro Techniques OR in vitro) in Title Abstract Keyword - (Word variations have been searched)	32
Embase	('tooth'/exp OR tooth OR 'teeth'/exp OR teeth OR 'enamel'/exp OR enamel OR 'dentin'/exp OR dentin) AND ('radiotherapy'/exp OR radiotherapy OR 'irradiation'/exp OR irradiation OR 'radiation therapy'/exp OR 'radiation therapy' OR (('radiation'/exp OR radiation) AND ('therapy'/exp OR therapy)) OR 'radiation treatment'/exp OR 'radiation treatment' OR (('radiation'/exp OR radiation) AND ('treatment'/exp OR treatment)) OR 'oral cancer treatment' OR (oral AND ('cancer'/exp OR cancer) AND ('treatment'/exp OR treatment)) OR 'ionizing radiation'/exp OR 'ionizing radiation' OR (ionizing AND ('radiation'/exp OR radiation))) AND ('dental bonding'/exp OR 'dental bonding' OR (('dental'/exp OR dental) AND ('bonding'/exp OR bonding)) OR 'bond strength'/exp OR 'bond strength' OR (('bond'/exp OR bond) AND ('strength'/exp OR strength)) OR 'marginal discoloration' OR (marginal AND ('discoloration'/exp OR discoloration)) OR 'microleakage'/exp OR microleakage OR debonding OR 'interfacial fracture toughness' OR (interfacial AND ('fracture'/exp OR fracture) AND ('toughness'/exp OR toughness)) OR 'marginal adaptation' OR (marginal AND ('adaptation'/exp OR adaptation))) AND ('in vitro techniques'/exp OR 'in vitro techniques' OR (in AND vitro AND techniques) OR 'in vitro'/exp OR 'in vitro' OR (in AND vitro))	530
OpenGrey through DANS	(Tooth OR teeth OR enamel OR dentin) AND (Radiotherapy OR irradiation OR radiation therapy OR radiation treatment OR oral cancer treatment OR ionizing radiation) AND (Dental Bonding OR bond strength OR marginal discoloration OR microleakage OR debonding OR interfacial fracture toughness OR marginal adaptation) AND (In Vitro Techniques OR in vitro)	26
PubMed	(Tooth OR teeth OR enamel OR dentin OR "Dental Enamel"[Mesh] OR "Dentin"[Mesh]) AND (Radiotherapy OR irradiation OR radiation therapy OR radiation treatment OR oral cancer treatment OR ionizing radiation OR "Adhesives/radiation effects"[Mesh] OR "Bisphenol A-Glycidyl Methacrylate/radiation effects"[Mesh] OR "Composite Resins/radiation effects"[Mesh] OR "Dental Enamel/radiation effects"[Mesh] OR "Dentin/radiation effects"[Mesh] OR "Dentin-Bonding Agents/radiation effects"[Mesh] OR "radiotherapy" [Subheading] OR "radiation effects" [Subheading]) AND (Dental Bonding OR bond strength OR marginal discoloration OR microleakage OR debonding OR interfacial fracture toughness OR marginal adaptation OR "Dental Bonding"[Mesh]) AND ("In Vitro Techniques"[Mesh] OR "in vitro")	380
Web of Science	(Tooth OR teeth OR enamel OR dentin) AND (Radiotherapy OR irradiation OR radiation therapy OR radiation treatment OR oral cancer treatment OR ionizing radiation) AND (Dental Bonding OR bond strength OR marginal discoloration OR microleakage OR debonding OR interfacial fracture toughness OR marginal adaptation) AND (In Vitro Techniques OR in vitro)	328

Table S2: RoBDEMAT tool used to assess the risk of bias of included studies

Domain	Sources of bias	RoBDEMAT signaling question(s)	Answer
D1	(1.1) Control group	Did the study employ one or more control groups (positive or negative or existing standard) in its experimental design?	
	(1.2) Randomization of samples	Was randomization adequately carried out and reported?	
	(1.3) Sample size rationale and reporting	Did the study provide a rationale and justification for the sample size chosen or feature an a priori power analysis?	
D2	(2.1) Standardization of samples and materials	Were samples and material choice/employment standardized according to the aim of the study?	
	(2.2) Identical experimental conditions across groups	Were the storage, experimental or treatment conditions standardized across samples and materials?	
D3	(3.1) Adequate and standardized testing procedures and outcomes	Were testing procedures and outcome(s) measure(s) explained or defined in sufficient detail to allow reproducibility and critical appraisal?	
	(3.2) Blinding of the test operator	Was the test operator blinded to the different experimental groups?	
D4	(4.1) Statistical analysis	Was the statistical analysis adequate and reported in sufficient detail?	
	(4.2) Reporting study outcomes	Are all relevant outcome data, expected to be reported, available in sufficient detail?	

Each signaling question should be answered as either "sufficiently reported/adequate" (1), "insufficiently reported" (2), "not reported/not adequate" (3), or "not applicable" (4).
